# Lapatinib Plus Local Radiation Therapy for Brain Metastases From HER-2 Positive Breast Cancer Patients and Role of Trastuzumab: A Systematic Review and Meta-Analysis

**DOI:** 10.3389/fonc.2020.576926

**Published:** 2020-11-06

**Authors:** Muhammad Khan, Zhihong Zhao, Sumbal Arooj, Tao Zheng, Guixiang Liao

**Affiliations:** ^1^ Department of Radiation Oncology, Shenzhen People’s Hospital, The First Affiliated Hospital of Southern University of Science and Technology, Shenzhen, China; ^2^ Department of Oncology, First Affiliated Hospital of Anhui Medical University, Hefei, China; ^3^ Department of Nephrology, Shenzhen People’s Hospital, Second Clinical Medicine Centre, Jinan University, Shenzhen, China; ^4^ Department of Biochemistry, University of Sialkot, Sialkot, Pakistan

**Keywords:** lapatinib, stereotactic radiosurgery (SRS), brain metastases (BM), overall survival (OS), radiation necrosis (RN), human epidermal growth factor receptor-2 (HER-2) positive breast cancer

## Abstract

**Background:**

Intracranial activity of lapatinib has been demonstrated in several studies in patients with human epidermal growth factor receptor-2 positive breast cancers (HER-2+ BC). Stereotactic radiosurgery (SRS) has been increasingly used as the local therapy for brain metastases in breast cancer patients. Increased objective response rate was observed for lapatinib plus whole brain radiotherapy (WBRT) is such patients with high toxicity.

**Objective:**

We seek to obtain clinical evidence of synergistic efficacy of lapatinib in combination with radiation therapy, in particular, SRS.

**Materials and methods:**

We carried out a comprehensive research using the following databases: PubMed; Medline; EMBASE; Cochrane library. These databases were searched until 10 June 2020. PRISMA guidelines were followed step by step for carrying out this systematic review and meta-analysis. Review Manager v 5.4 software was used for statistical evaluation of data.

**Results:**

Overall 6 studies with 843 HER-2 positive breast cancer patients (442 HER-2 amplified disease, 399 luminal B disease) were included in this systematic review and meta-analysis. A total 279 patients had received lapatinib in addition to HER-2 antibody (trastuzumab) plus/minus chemoradiotherapy, while 610 patients had received trastuzumab-based management or only chemoradiotherapy. Lapatinib-based management of BM was associated with significant increase in overall survival (HR 0.63 [0.52, 0.77], p < 0.00001). Combination of the two (trastuzumab plus lapatinib) was associated with increased survival advantage compared to each agent alone (0.55 [0.32, 0.92], p = 0.02). SRS in combination with lapatinib was associated with increased local control (HR 0.47 [0.33, 0.66], p = 0.0001). Ever use of lapatinib with SRS was associated an increased survival as reported in two studies (Shireen et al.: 27.3 vs. 19.5 months, p = 0.03; Kim et al.: 33.3 vs. 23.6 months, p = 0.009). Kim et al. also revealed significant increase in intracranial activity with concurrent lapatinib reporting 57% complete response compared to 38% (p < 0.001) and lower progressive disease rate of 11 vs. 19% (p < 0.001). Risk of radiation necrosis was decreased with lapatinib use.

**Conclusions:**

Lapatinib has shown intracranial activity and yielded better survival for HER-2+ BC patients with BMs. SRS in combination with ever use of lapatinib had better local control and were associated with better survival. Radiation necrosis risk was reduced with the use of lapatinib.

## Introduction

Breast cancer is the leading type of cancer in women according to the estimated number of new cases in 2020 (1–3). An estimated 276,480 new cases of breast cancer will be diagnosed with an estimated 42,170 breast cancer deaths will occur in women (1, 2). A surge of 0.3% per year from 2007 to 2016 has been observed in invasive breast cancer incidence rate (1, 2). Mortality rate, on the other hand, has been declined by 40% from 1989 (33.2/100,000) to 2017 (19.8/100,000) (1, 2). These improvements in management of breast cancer reflects the advancements in screening and awareness and treatment paradigm (1–6). Traditionally, breast cancer patients are managed with surgery, chemotherapy and radiation therapy (3–6). Advancements in the molecular understanding have added newer targeting agents to the treatment regimen that target various breast cancer subtypes according to the receptors expressed by breast cancer cells such as Luminal A (ER/PR positive/HER2 negative), HER2 (HER2 positive/ER/PR negative), luminal B (triple positive), and basal (triple negative) (6–8). In the case of luminal B and HER-2 amplified disease, these advancements include: the endocrine therapy for ER/PR positive breast cancer, monoclonal antibodies (trastuzumab and pertuzumab), multi-kinase inhibitors (lapatinib, neratinib, and tucatinib), and antibody-drug conjugates (ADC) targeting the HER-2 overexpressing breast cancers (6–12). Furthermore, molecular targeted agents targeting various subsequent intracellular signaling pathways such as inhibitors of cyclin-dependent kinases 4 and 6 (CDK4/6), phosphatidylinositol 3-kinase (PI3K), protein kinase B (Akt), and mammalian target of rapamycin (mTOR) pathways have also been developed to enhance the outcomes (6–9). These advancements have led to rapid improvement in the outcome for breast cancer patients in recent years as also manifested by the decrease in death rate of 1.3% per year from 2013 to 2017. 5- and 10-year survival rates for women with invasive breast cancer are 91 and 84%, respectively (1, 2).

Broadly, breast cancer is categorized into *in situ* carcinoma and invasive carcinoma (3, 4, 13, 14). Invasive breast cancer invades or metastasize to other parts or organs of the body and is composed of mainly two categories: infiltrating lobular carcinoma (ILC) and infiltrating ductal carcinoma (IDC) (13, 14). ILC accounts for 10–15%, while IDC makes up the 80% of breast cancer diagnosis (7, 8). Breast cancer is the second most frequent cancer to cause brain metastases (15–25%) after lung cancer (40–50%) (15). Fractionated WBRT has only managed limited local control (median intracranial failure: 3–5 months) and median survival time around 3–4 months (16, 17). SRS alone or in combination with WBRT, surgery, or both were superior to WBRT alone in prolonging the survival in breast cancer patients with brain metastases (18). SRS alone has reported impressive local control rates between 90 and 94% and median survival between 10 and 16 months (16). Hence, SRS alone has emerged as main treatment for brain metastases alone or in combination (19, 20). However, various breast cancer subtypes also have responded distinctly from the prognostic point of view to surgical and radiotherapeutic management (21–24). Sperduto et al. have revealed luminal A and luminal B treated with surgery, SRS, and WBRT alone or in various combinations were superior according to time from primary diagnosis to brain metastases (TPDBM) (LA: 54.4, LB: 47.4, HER2: 35.8, and B: 27.5, months (p < 0.01), and survival from primary diagnosis (PD survival) (LA: 54.4, LB: 47.4, HER2: 35.8, and B: 27.5 months, p < 0.01) (LA: 72.7, LB: 90.3, HER2: 66.4, and B: 39.6 months, p < 0.01) (23). On the other hand, patients with HER2 positive patients (luminal B and HER2 positive) reported better survival from the time of BM diagnosis (BM survival) compared to other two types (LB: 22.9, HER2: 17.9, LA: 10, and B: 7.3 months, p < 0.01) (23). Similarly, in a separate retrospective study (n-131), breast cancer patients were treated with SRS alone for their brain metastases reported a trend towards better survival in patients with HER2 positive subgroups (LB: 26, HER2: 23, LA: 16, and B: 7 months, p < 0.001) (24). These outcomes suggest HER2 overexpressing BC patients with brain metastases are placed at better prognosis if treated with SRS alone or in combination with other treatment regimens (surgery, WBRT).

Human epidermal growth factor receptor 2 (EGFR-2/HER-2) also known as erythroblastic leukemia viral oncogene homolog 2 (ERBB2) protein, is overexpressed by around 20 to 30% of breast cancers (25, 26). HER2-positive and triple-negative status were identified as risk factors for the development of BM (21). About one third of the HER-2 overexpressing metastatic breast cancer patients develop brain metastases (27). Before the advent of targeted therapy and chemotherapeutic agents against HER-2 positive breast cancer, HER-2 positive status was associated with worst overall survival compared to HER-2 negative BC (28, 29). Trastuzumab, an anti-HER-2 monoclonal antibody, have been developed and approved for HER2 positive metastatic breast cancer patients along with chemotherapy based on the results of phase I and phase II trials (9). Despite improvement in overall survival with trastuzumab, it is deemed ineffective against prevention of BM development and intracranial activity due to its heavy molecular weight (30–35). In fact, treatment with trastuzumab was related to higher BM incidence in these patients (32–35). It could be attributed to longer survival and better control of systemic disease. Moreover, 25% of patients relapse after adjuvant trastuzumab-based treatment for HER-2 BC patients (26). HER-2 positive metastatic breast cancer patients progressing on trastuzumab-based therapy were allowed to be treated with lapatinib, a dual tyrosine-kinase inhibitor of EGFR and HER2, in combination with capecitabine; FDA had approved the combo “lapatinib plus capecitabine” on March 13, 2007 (10). As opposed to trastuzumab, lapatinib is a small molecule and it is suggested it may penetrate BBB to have efficacy in the brain as well. Lapatinib was shown to prevent the development of brain metastases in HER-2 positive breast cancer patients (27, 36). Lapatinib as monotherapy had shown a modest intracranial activity in trastuzumab-pretreated patients with progressive CNS disease after radiotherapy (27, 37). A 20% intracranial response rate was observed in patients treated with lapatinib plus capecitabine combination (37).

There is growing interest in combining the radiation therapies and targeted or immune therapies in order to seek synergism between the treatments. Hence, lapatinib with WBRT was investigated for treating HER-2 positive brain metastases. Though, an objective response rate of 79% was achieved, the combination was not feasible due to safety concerns (38). Similarly, several retrospective studies have also investigated the concurrent use of lapatinib with radiation therapy (39–44). The results are contradicting with no established recommendations in this direction. Therefore, we have undertaken this systematic review and meta-analysis in order to synthesize a meta-outcome for a better clinical perspective.

## Materials and Methods

This “systematic review and meta-analysis” was undertaken according to the guidelines provided by the “Preferred Reporting Items for Systematic Reviews and Meta-Analyses” (PRISMA) (45). A protocol of this study is registered on PROSPERO: CRD42020191615.

### Inclusion Criteria

#### Patients and Study Types

Comparative studies involving breast cancer patients with brain metastases treated with lapatinib in conjunction with SRS/WBRT or SRS only. Comparative studies with any experimental design (retrospective, prospective, clinical trial, and randomized controlled trials) were allowed for selection.

#### Types of Interventions

Lapatinib, a dual tyrosine kinase inhibitor interrupting both HER2/neu and epidermal growth factor receptor pathways, in conjunction with SRS/WBRT was labeled as the “Experimental intervention group” and SRS/WBRT only as the “Control intervention group”.

#### Outcomes of Interest

Efficacy outcomes such as survival, brain control, and brain objective responses, and safety outcomes mainly the adverse events related to treatment. Overall survival was characterized as the primary outcome of interest while all other outcomes were of secondary interest to our analysis.

### Search Strategy

#### Databases

We carried out a comprehensive research using the following databases: PubMed; Medline; EMBASE; Cochrane library. These databases were searched until 10 June 2020. Various key search terms were used with English language restriction. As well, various studies’ references were search for relevant studies.

#### Study Selection

Studies while screening for titles and abstracts were incorporated into the Endnote X9 software for organizing, further screening, and scrutiny. Duplicate studies were removed after proper assessment. Studies were selected or rejected according to inclusion and exclusion criteria by two independent reviewers. Full text assessment was carried out for selected studies. Any disagreement was resolved by mutual consensus between reviewers.

#### Data Extraction

Data was extracted and incorporated into the data collection form provided by Cochrane organization named as “The Cochrane Collaboration Data Collection form-RCTs and non-RCTs”. Extracted data included various studies’ attributes and patients’ baseline characteristics. Attributes of the studies included study design, number of participants, year of publication, time period, treatment regimens, main outcomes for the whole study population, and median follow ups. Baseline characteristics of the patients included age, sex, performance status, number of brain metastases, previous therapies, subsequent systemic, or other therapies. Comparative outcome data was also recorded such overall survival, local and distant control, and safety measures.

#### Assessment of Risk for Bias

Downs and Black checklist was used for risk of bias assessment (46). Downs and Black checklist developed for the assessment of the methodological quality of non-randomized interventional studies consists of 27 questions covering four aspects of quality assessment namely reporting, external validity, internal validity (bias and confounding), and statistical power. A single point is given for each question if the answer is in affirmative and 2 points in case of one question in reporting section. Reporting section consists of 10 questions, and external validity of 3 questions. Internal validity is comprised of 13 questions and involves 2 sections; bias, and confounding. We used the modified version of Downs and Black checklist in which statistical power question is answered as yes or no with a single point score as compared to original checklist in order to simplify calculation and avoid ambiguity (47). Gradation was assigned according to score as “excellent” (24–28 points), “good” (19–23 points), “fair” (14–18 points) or “poor” (<14 points).

#### Measurement of Treatment Effect and Data Synthesis

Hazard ratios for survival time were recorded if given in the study or extracted from K-M curves using Digital Equalizer software and the methods described in the study by Tierney et al. for incorporating summary time-to-event data into meta-analysis (48). Similar procedure was followed for local control rates. Pooling of hazard ratios was done using RevMan v 5.4 software (49, 50). Inverse variance or M-H method was used for pooling hazard ratios or odds ratios, respectively. Fixed effects model or random effects model was adopted according to the level of heterogeneity (I^2^). Heterogeneity more than 50% was considered as moderate and criteria for adopting random effects model (51). Significance level was set at p value less than 0.05.

## Results

**Figure 1:**
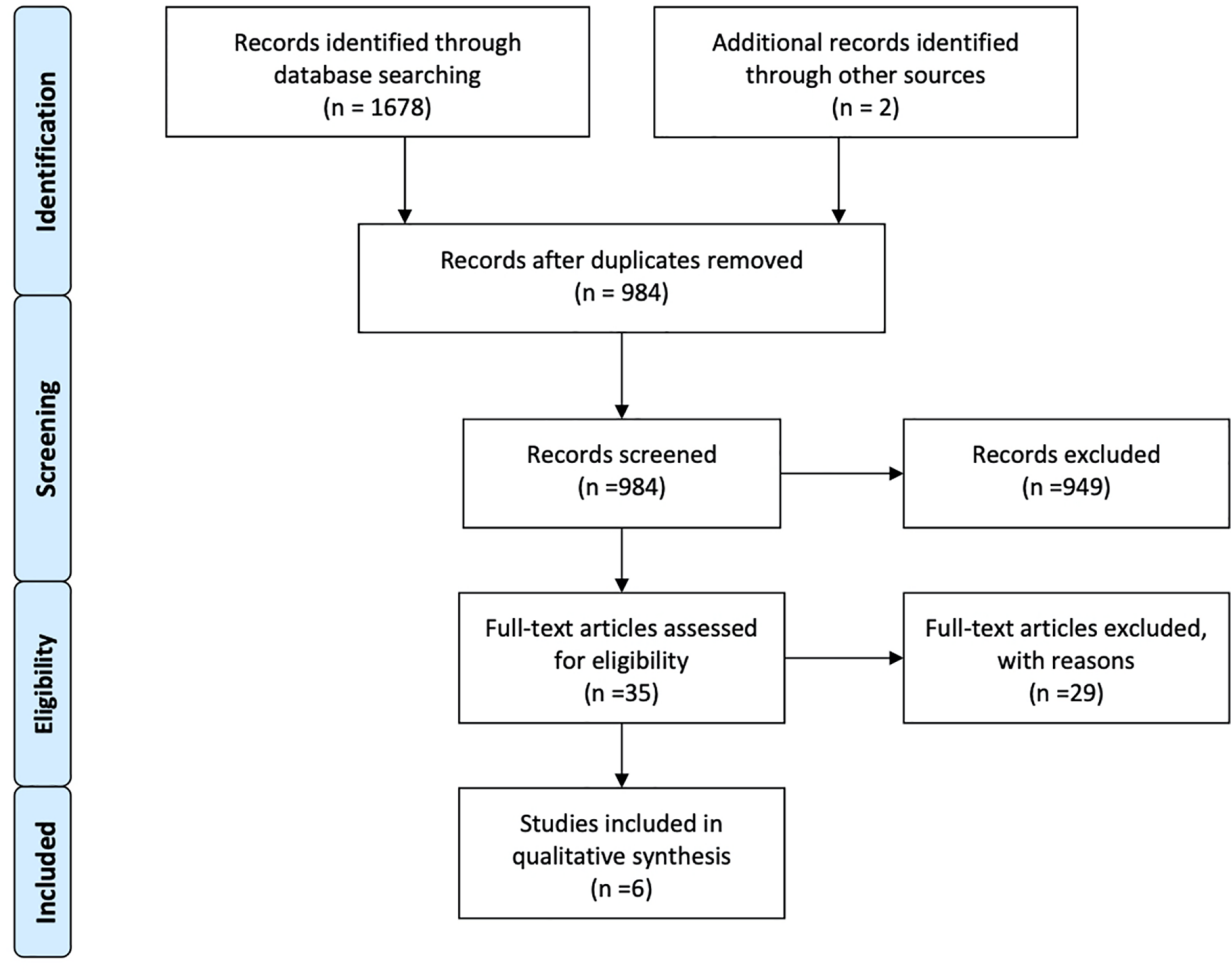
PRISMA flow diagram of search strategy and study selection.

Overall six studies were identified meeting the inclusion criteria through robust research strategy and study selection process (39–44) (**Figure 1**). A total of 843 HER-2 positive breast cancer patients with brain metastases treated with radiation therapy as local treatment plus/minus chemotherapy and anti-HER2 therapy (trastuzumab/lapatinib) over a time period between 1997 to 2015 constituted the participants of this meta-synthesis. 442 had HER-2 amplified disease while 399 had luminal B disease. A total of 279 patients had been treated with lapatinib plus/minus anti-HER2 antibody, and 610 patients were either treated with ant-HER2 antibody (mainly trastuzumab) or in some cases without any anti-HER2 therapy (n = 227). Patients with luminal B disease were also treated with hormone therapy. SRS as local therapy plus/minus WBRT was mainly used in all studies (n = 404). While WBRT alone was used in three studies only, it constituted the main treatment option according to the number of patients (n = 484) (39, 40, 42). All the included studies were retrospective in nature and classified as class III evidence (39–44). The studies were graded as “fair” or “good” after quality assessment as illustrated in **Table 1**. All the studies scored low on selection bias and power (39–44). The studies graded fair had also scored a bit lower on reporting assessment (39, 41, 43).

**Table 1:** General characteristics of the included studies.

Studies	Time period	Number	Lesions	Radiation therapy	Median OS	ORR	Local control	Radiation necrosis	QA
**Bartsch et al. (** 39 **).**	1998–2010	80	–	WBRT SRS	10 months (95% CI: 6.31–13.69)	–	–	–	17
**Yap et al. (** 40 **)**	2006–2008	280	–	WBRT SRS	10.9 months (95% CI 9.0–11.9)	–	–	–	19
**Yomo et al. (** 41 **).**	2009–2012	40	382	SRS	16.6 months (95 % CI: 13.5–29.8)	–	–	–	16
**Miller et al. (** 42 **).**	1998–2014	233	479	WBRT SRS Surgery	–	–	LF:15.4% (95% CI: 9.7–21.1%) DF:23%	5.6%	20
**Kim et al. (** 43 **).**	2005–2014	84	487	SRS		78% [−100%, +125%]		6%	18
**Shireen et al. (** 44 **).**	1997–2015	126	479	SRS	23.5 months (95% CI 17.9–27.8 months)		LF:15.4% (95% CI: 9.7–21.1%)	5.6%	19

SRS, stereotactic radiosurgery; OS, overall survival; LC, local control; LF, local failure; DC, distant control; DF, distant failure; WBRT, whole-brain radiation therapy; RN, radiation necrosis; ORR, objective response rate; MST, median survival time.

### Baseline Characteristics of the Patients

Baseline characteristics of the patients were mostly matched without any significant differences except for age, KPS, and prior local therapy. Shireen et al. study (n = 126) lapatinib receiving participants (n = 47) were younger in age (p = 0.025), and had partially better KPS score (p = 0.091) (44). Participants in the four arms of Yap et al. study also had age differences (0.089) (40). While in the study by Kim, et al., concurrent lapatinib group patients had received prior WBRT for a greater number of lesions (80 vs. 53%, p < 0.001), and SRS boost to WBRT was also received by fewer lesions (2 vs. 10%, p = 0.005). As well, a greater number of lesions treated with SRS alone had also been resected previously (2 vs. 19%, p < 0.001) (43). Except for these differences, no other baseline characteristics differences were noticed such as extracranial disease, number of brain metastases, RPA classification and DS-GPA categorization (39–44). General characteristic of the studies and patients are outlined in **Tables 1**, **2**.

**Table 2:** Baseline characteristics of patients and main outcomes of interest.

Characteristics/studies	Bartsch et al. (39)	Yap, et al. (40)	Yomo, et al. (41)	Miller et al. (42)	Kim et al. (43)	Shireen et al. (44)	This study
**Comparative groups**	Lapatinib/no lapatinib	Lapatinib/no lapatinib	Lapatinib/no lapatinib	Lapatinib/no lapatinib	Lapatinib/no lapatinib	Lapatinib/no lapatinib	Lapatinib/no lapatinib
**No. of patients**	80 (15/65)	280 (58/222)	40 (24/16)	233 (89/187)	84 (43/41)	126(47/79)	843 (276/610)
**Age (median [range]) (years)**	53 (28–77)	52 (25–81) (p = 0.084)	58.5 (37–72)	52 (23–80) 53 (28–87)	52 [31–84]	54 (31–84) (p = 0.025)	
**HER2+**	42	159	40	99	51	51	442
**HR**	38	119		134	33	75	399
**Lapatinib +/- HER2 mAb**	15	30	24	89	43	47	248
**Transtuzumab**	28	56	34	187	64	55	424
**Both**	–	28	–	–	–	–	28
**No anti-HER2 therapy**	37	166	–	–	–	24	227
**Concurrent lapatinib**	–	–	–	–	18	24	42
**SRS +/- WBRT**	40	32	40	82	84	126	404
**WBRT**	40	251	–	193	–	–	484
**Surgery**	–	35	–	35	–	–	70
**Median OS**	-Anti-HER2: HR: 0.29; 95% CI: 0.16–0.54; p < 0.001 Lapatinib: HR: 0.279; 95% CI: 0.1–0.76; p = 0.012	-Anti-HER2: MST: 18.5 vs. 5.7, p < 0.001 -Lapatinib: Both agent: 25.9 (18.5–30.1); Lp alone: 21.4 (12.5–27.1); T alone: 10.5 (8.3–17.7); No anti-HER2: 5.7 (4.2–8.9), p < 0.001	MST: 19.5 vs. 15, p = 0.530	MST: 21.1 vs. 15.4 months; p = 0.03	-Concurrent: MST: 40.4 vs. 25.1 months (p = 0.155) -Ever use: MST: 33.3 vs. 23.6 months (p = 0.009	MST: 27.3 vs. 19.5 months, p = 0.03	
**Objective response rate (ORR)**	–	–	–	–	-Concurrent: CR: 35 vs. 11%, p = 0.008 ORR: (CR + PR, 75 vs. 57%, *P* = 0.121) PD: 25% vs. 43%, p = 0.121 MBOR: 69 vs. 54% (p = 0.037) -Lesion-specific BOR: median 100 vs. 70% reduction, p < 0.001 CR: 57 vs. 38%, p < 0.001 PD: 11 vs. 19%, p < 0.001	–	
**Local control** **(12-m cumulative incidence)**	–	–	LC: 86 vs. 69%, p < 0.001	LF: 15.1 vs. 5.7%, p < 0.001) (only SRS)	LF: 12 vs. 19%, p = 0.071 (concurrent)	LF: 15.1 vs. 5.7%, p < 0.001)	
**Distant control**	–	–	–	DF: 9.2 vs. 18.3%, p = 0.08	DF: 48 vs. 49%, p = 0.91 (concurrent)	–	
**Radiation necrosis**	–	–	–	RN: 1.3 vs. 6.3%, p = 0.001	RN: 1.0 vs. 3.5%, p = 0.134 (concurrent)	RN: 1.3 vs. 6.3%, p = 0.001	

SRS, stereotactic radiosurgery; OS, overall survival; LC, local control; LF, local failure; DC, distant control; DF, distant failure; WBRT, whole-brain radiation therapy; RN, radiation necrosis; CR, complete response; PD, progressive disease; ORR, objective response rate; MBOR, median best objective response; BOR, best objective response; PR, partial response; MST, median survival time; HR, hazard ratio; HER-2, human epidermal growth factor receptor 2; mAb, monoclonal antibody.

### Overall Survival

Overall survival was analyzed at several levels depending on the status of lapatinib use (ever/concurrent), combination with trastuzumab, and the type of radiation therapy received (WBRT/SRS).

#### Lapatinib vs. Non-Lapatinib-Based Therapy

**Figure 2:**
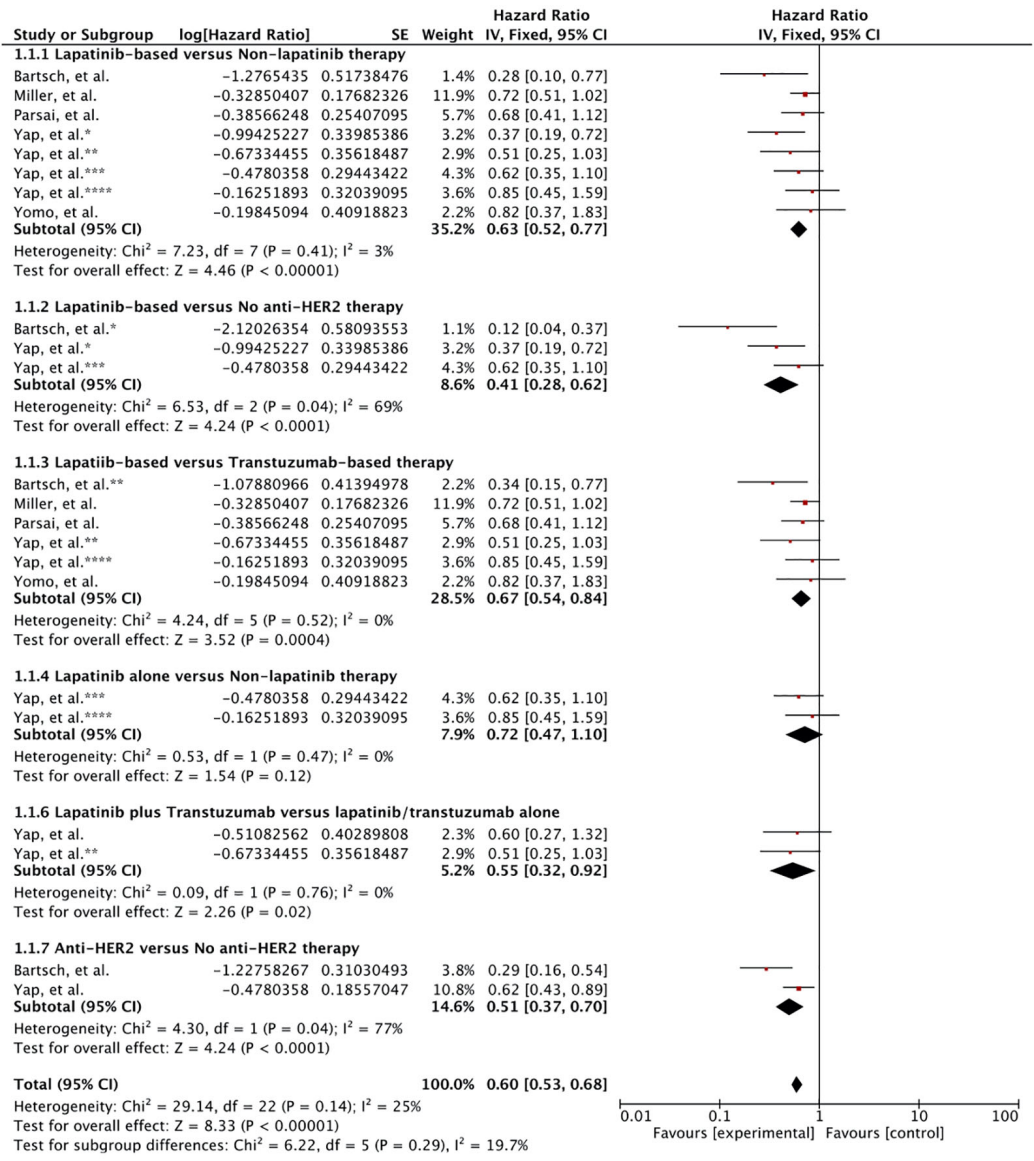
Forest plot of meta-analysis of overall survival (OS) for treatment comparison (Lapatinib versus non-lapatinib-based therapy) in the management of brain metastases from HER-2 positive breast cancer.

First, lapatinib (ever used or given concurrently) plus/minus trastuzumab (given sequentially or concurrently) plus/minus chemotherapy with local therapy for BM compared to BM patients who had never used lapatinib and may or may not have utilized trastuzumab and cytotoxic chemotherapy but had undergone local surgical and/or radiation therapy (WBRT and SRS) for brain metastases. Patients receiving lapatinib concurrently or any time during the course of their disease for BM was associated with improved survival compared to patients without any lapatinib use. All the six studies reported survival outcome involving 843 patients. Meta-analysis of OS revealed a significant improvement in overall survival based on the results from five studies (HR 0.63 [0.52, 0.77], p < 0.00001) (**Figure 2**; Outcome 1.1.1) (39–42, 44). Kim et al. also revealed a significant increase in survival benefit (MST: 33.3 vs. 23.6 months, p = 0.009) for patients ever (n = 43) receiving lapatinib as compared to never (n = 41) (43).

### Survival Subgroup Analysis

#### Lapatinib vs. No Anti HER2 Therapy

Two studies had reported the outcome for patients receiving lapatinib compared to patients that had received no anti-HER2 therapy at all. Pooled hazard ratio revealed that any use of lapatinib was significantly better in comparison to lack of anti-HER-2 therapy (HR 0.41 [0.28, 0.62], p < 0.0001) (**Figure 2**; Outcome 1.1.2) (39, 40).

#### Lapatinib vs. Trastuzumab-Based Therapy

In several studies, the control group were exposed trastuzumab. In this case, lapatinib use was associated with significant increase in survival compared to the use of trastuzumab as the only anti-HER2 therapy (HR 0.67 [0.54, 0.84], p = 0.0004) (**Figure 2**; Outcome 1.1.3) (39–42, 44).

#### Lapatinib Alone vs. No Lapatinib Therapy

Yap et al. also reported the comparative outcome for patients receiving only lapatinib as anti-HER2 therapy (40). Meta-analysis revealed no survival advantage (HR 0.72 [0.47, 1.10], p = 0.12) (**Figure 2**; Outcome 1.1.4). However, this result was mainly based on the results from single study (40).

#### Lapatinib Plus Trastuzumab vs. Lapatinib/Trastuzumab Alone

Moreover, use of both lapatinib plus trastuzumab sequentially or concurrently was shown to be superior compared to each given alone (0.55 [0.32, 0.92], p = 0.02) (**Figure 2**; Outcome 1.1.5) (40). Nonetheless, the effect was much better compared to trastuzumab alone (p = 0.055) than lapatinib alone (p = 020) as revealed by Yap et al. (40).

#### Anti-HER2 vs. No Anti-HER2 Therapy

At last, use of anti-HER2 therapy versus no anti-HER2 therapy was investigated. Overall, the use of anti-HER2 therapy comprising lapatinib and trastuzumab prolonged the survival for breast cancer patients expressing HER-2 with brain metastases (0.51 [0.37, 0.70], p = 0.0001) (**Figure 2**; Outcome 1.1.6) (39, 40). The result was based on outcomes from two studies (39, 40).

#### Lapatinib Plus SRS Only as Local Therapy

As a subgroup, we also evaluated the results involving SRS as the only therapy for BM and use of concurrent lapatinib. Shireen et, al. revealed a significant survival advantage for patients with any use of lapatinib (MST: 27.3 vs. 19.5 months, p = 0.03) (44). Patients in the study by Yomo et al. also had involved only SRS (41). Lapatinib-based therapy was not associated survival advantage (MST: 19.5 vs. 15.0 months, p = 0.530). However, low number of patients with high cross over was reported in this study. Though, Kim et al. revealed survival advantage with any use of lapatinib (MST: 33.3 vs. 23.6 months (*P* = 0.009) but concurrent use of lapatinib given with SRS (n = 18) compared to SRS alone (n = 66) was not associated with survival surge (MST: 40.4 vs. 25.1 months, p = 0.155) (43). Kim et al. also evaluated survival difference for patients using concurrent lapatinib versus any use of lapatinib within the group of 43 patients who had ever used lapatinib. There was no survival difference (40.4 vs. 33.3 months, p = 0.775).

### Objective Response Rate

Only one study evaluated the objective response rate for treatment difference (43). Lesion-specific best objective response was superior in patients receiving concurrent lapatinib compared to SRS alone (100 vs. 70% reduction, p < 0.001). Complete response was 57% with concurrent lapatinib compared to 38% (p < 0.001) as well as lower progressive disease rate of 11 vs. 19% (p < 0.001). Median objective response rate at 6-, and 12-month were: 100 vs. 60% (p < 0.001), and 100 vs. 71% (p < 0.001).

Kim et al. also investigated timing of lapatinib intervention and objective response. Overall response and best objective response were generally better when the intervention timing was closer to SRS induction. Median objective response was 100% when lapatinib was initiated concurrently (n = 132; CR: 57%, PD: 11%) or within 3 months of SRS induction (n = 150; CR: 55%, PD: 14%). For patients receiving prior to SRS, objective response was 77% (n = 94; CR: 40%, PD: 7%), while it was 78% (n = 46; CR: 43%, PD: 15%) for patients receiving it in 3 to 6 months and 85% (n = 75; CR: 48%, PD: 31%) for patient using it after more than 6 months. Patients never using lapatinib reported the least objective response of 54% (n = 111; CR: 22%, PD: 23%).

### Local Failure

**Figure 3:**
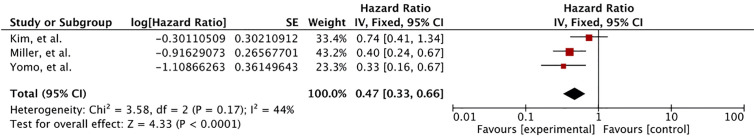
Forest plot of meta-analysis of local control (LC) for treatment comparison (Lapatinib plus SRS versus SRS alone) in the management of brain metastases from HER-2 positive breast cancer.

Local control was significantly increased with SRS plus lapatinib based on the meta-analysis of three studies (HR 0.47 [0.33, 0.66], p = 0.0001) (41–43) (**Figure 3**). Two of these studies involved concurrent use of lapatinib with SRS indicating lapatinib given concurrently improves local control comparatively better.

### Distant Failure

Distant failure was reported in two studies for treatment difference (42, 43). Comparatively lower rate of distant failure was observed with use of TKIs as 12-month cumulative incidence of DF was 9.2% (95% CI: 0.0–19.4%) with TKIs use in comparison to 18.3% (95% CI: 14.8–21.8%) without the use of TKIs. However, this difference was not significant (p = 0.08) (42). In the study by Kim et al., no such difference was observed (43). 12-month cumulative incidence was 48% (95% CI: 28–68%) with concurrent lapatinib as opposed to 49% (95% CI: 40–58%) without concurrent lapatinib (p = 0.91).

### Radiation Necrosis

Two studies reported radiation necrosis rate revealing a lower rate for concurrent lapatinib and SRS (42, 43). In the study by Miller et al., concurrent HER2/lapatinib with SRS was associated with lower 12-month cumulative incidence of radiation necrosis (1.3 vs. 6.3%, p = 0.001) (42). Shireen et al. further revealed the 6-month (0.0 vs. 4.1%), 12-month (1.3 vs. 6.3%), and 24-month (1.9 vs. 8.2%) cumulative incidences of radiation necrosis for concurrent lapatinib (44). Kim et al. also identified no association of concurrent lapatinib with increasing rates of radiation necrosis (43). 12-month cumulative incidence rate of grade 2+ RN among patients with or without concurrent lapatinib was similar (1.0 vs. 3.5%, p = 0.134). Concurrent lapatinib was also not associated with increase rates of RN among larger lesions (<1.5cm) despite an association between RN and increasing volume of lesion in the participants (0.0 vs. 4.5%, p = 0.39).

### Publication Bias

**Figure 4:**
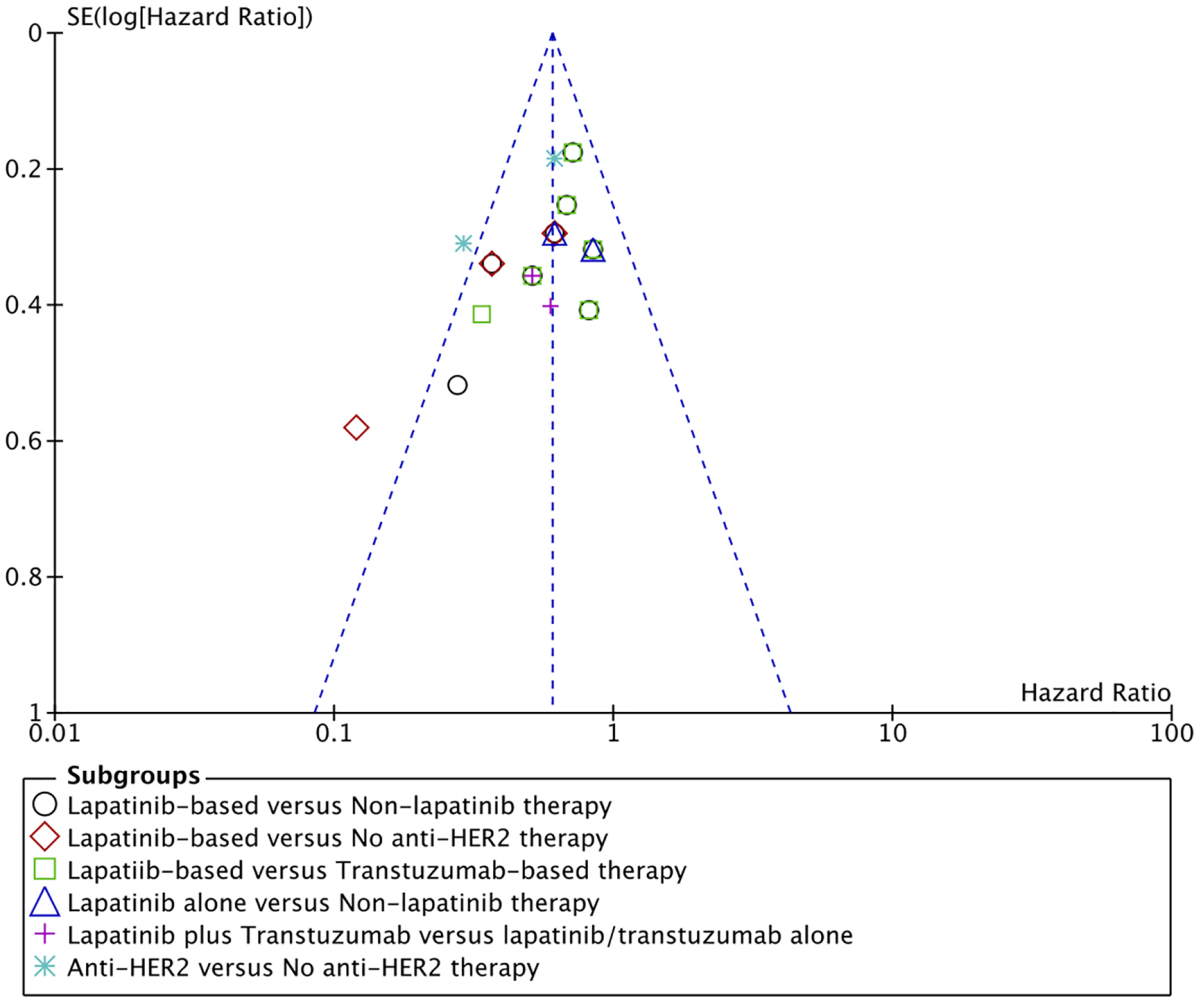
Funnel plot of publication bias assessment for overall survival.

Funnel plot for overall survival was used for assessment of publication bias. All results were within the 95% CI except for one comparison (**Figure 4**). In that comparison, lapatinib-based management was compared to patients managed with radiotherapy alone without any chemotherapy (39). Such drastic difference between the treatments made this comparison comparatively unique.

## Discussion

Management of brain metastases has been improving over the years with the introduction of newer targeting agents as well as advancements in the radiation and chemotherapy fields. SRS is being preferred over other local therapies or combined with other agents such as WBRT and surgery for treating BM including BC BM (52). Johnson et al. reviewed 737 BM patients between 2000 and 2013 who had undergone upfront SRS for BMs, of which 167 had also received targeted agents concurrently or within 30 days of SRS (53). Overall, the use of targeted agents was associated with improved survival (65 vs. 30% at 12 months, p < 0.0001), local control (94 vs. 90% at 12 months, p = 0.06), distant brain control (32 vs. 18% at 12 months, *p* = 0.0001), and freedom from WBRT (88 vs. 77% at 12 months, p = 0.03). On the hand, in a similar retrospective study, in which 1650 BM patients treated with SRS plus/minus WBRT/surgery were investigated for concurrent use of targeted therapy (54). Use of targeted agents concurrently with SRS was not associated with survival on multivariate analysis (HR 0.90, 95% CI 0.78–1.03, p = 0.11). Nonetheless, breast cancer patients had achieved significant survival advantage with the use of concurrent targeted agents (trastuzumab and lapatinib) (18.2 vs. 13.8 months, p < 0.01). These outcomes suggest a rise in the use of concurrent targeted agents with radiation therapy. Here we have reviewed literature for comparative evidence regarding the combined use of radiation therapy and lapatinib for HER-2 positive breast cancer patients with brain metastases.

Our meta-analysis reveals a significant increase in overall survival for HER-2 positive patients with brain metastases receiving lapatinib-based management. In the non-lapatinib based therapy, majority of the patients were exposed to trastuzumab. Lapatinib use was associated with additive survival advantage against patients not receiving any anti-HER2 therapy at all. Likewise, survival benefit was maintained in comparison to patients receiving trastuzumab-based management. This result is in line with the data from Metro et al. study (55). HER-2 positive BC patients with brain metastases treated with lapatinib plus capecitabine were associated with significantly higher median survival as opposed to trastuzumab-based therapies (MST: 27.9 vs. 16.7 months, p = 0.01). Radiotherapy was also delivered to majority (26/30) of patients but the duration between radiotherapy and LC initiation was equal to or greater than two months. In a study by Anatolian Society of Medical Oncology (ASMO), lapatinib plus capecitabine (n = 46) was revealed with significantly improved survival (MST: 19.1 vs. 12.0 months, p = 0.039) compared to patients receiving trastuzumab-based therapy (n = 65) for BM management (56). In this study, only a portion of patients had received radiosurgery (n = 33; 14/19) and neurosurgery (n = 16; 2/14). Similarly, a case report of 45 years old HER-2 BC patients with BM treated with RT followed by lapatinib plus capecitabine (with capecitabine for 10 months and with letrozole for 3 months) had also achieved a longer survival of 45 months (57). In this case, lapatinib plus capecitabine was re-inducted after brain relapse indicating successful reinduction of lapatinib in such patients.

Lapatinib alone after BM, on the other hand, was not associated with increased survival compared to no anti-HER2 therapy as well as trastuzumab alone. Even though, the result is based only on one study, it may indicate lapatinib inability to control extracranial disease. As it has been demonstrated in the study by Miller et al. that lapatinib was able to extend survival by 7 months for patients with extracranial disease (MST: 20 vs. 13 months, p = 0.14) as compared to 16 months for patients without extracranial disease (MST: 39 vs. 23 months, p = 0.05) (42). Instead, trastuzumab is associated with increased incidence of BM and neurologic cause of death in HER2 positive BC patients despite prolonging survival (30–35, 58). It has been suggested that better extracranial control with trastuzumab leads to prolong survival, hence, more patients are surviving to have BM, and it may also suggest trastuzumab ineffectiveness in the brain leading to more neurologic deaths (59). This fact could also be reflected in our results as combining the two were superior to each treatment alone particularly the trastuzumab in prolonging survival. Though, the majority of the patients in combined group received both agents sequentially (n = 22/28). The combination had also been shown in RCTs to prolong event-free survival and overall survival in comparison to each alone in HER-2 positive BC patients without BM (60, 61). In fact, pathological complete responses in combined group were higher as compared to each group alone (62–66). Rates of pCR were the lowest in lapatinib group (n = 154/414) compared to trastuzumab alone (n = 183/439) suggesting lower systemic activity of lapatinib (base/Bonn) (OR 0.85 [0.64, 1.14], p = 0.29) (62–66). The difference trended towards significance when data was restricted to 3 studies (L=53/205 vs. T=81/227; OR 0.69 [0.44, 1.06], p = 0.09) (62, 63, 66). Moreover, in head to head trials (TC vs. LC), contradicting results were obtained (67, 68). WJOG6110B/ELTOP trial failed to show any significant difference between the two treatments in patients pretreated with trastuzumab (67). CEREBEL trial, however, showed significant PFS (HR 1.30; 1.04–1.64, p = 0.021) and OS (HR 1.34; 0.95–1.90, p = 0.095) survival advantage for trastuzumab plus capecitabine lapatinib compared to lapatinib plus capecitabine (68). Difference in PFS was further increased when patients without any previous exposure to trastuzumab was considered (HR 1.70; 1.15–2.50). These outcomes suggest that lapatinib, though have intracranial activity, may still require trastuzumab to control the extracranial disease and vice versa in order to achieve best outcomes.

In our study, SRS alone was used in all the studies but WBRT, despite being used in only three studies, was the leading local therapy for BM according to the number of patients (39–44). Three studies had involved only SRS and 2 studies had used concurrent lapatinib with SRS (41, 43, 44). Administration of lapatinib with SRS or within five biological half-lives from the date of SRS was defined as concurrent lapatinib. Half-life elimination of lapatinib is 24 hours creating a window of 5 days before or after SRS for lapatinib induction (43). Ever use of lapatinib with SRS was significantly associated with survival as reported in two studies (43, 44). However, concurrent use was not revealed to have any survival advantage as demonstrated by Kim et al. (43). Even though, a trend towards significance in local control (p = 0.071) and significant higher rates of complete responses (35 vs. 11%, *P* = 0.008) were revealed in their study with concurrent lapatinib (43). Failure to report SRS advantage may come from the fact that number of patients were few (n = 18) in the concurrent lapatinib group (43). Meta-analysis of local control rates also revealed a significantly increased brain local control with application of SRS and lapatinib (41–43). This suggests survival advantage with SRS in combination with lapatinib could signify synergism between the two agents. As lapatinib is a small molecule and is assumed to cross BBB, however, its brain distribution was shown to be restricted by BBB (69, 70). Moreover, lapatinib alone has only obtained moderate intracranial responses (2.6–6%) (27, 37). Inability of lapatinib to affect distant control in the study of Kim, et al. also suggest its limited access to the brain as observed in case of other TKIs; VEGFR TKIs in RCC BM, and BRAF/MEK inhibitors in melanoma (43, 53, 71–74). These outcomes suggest that lapatinib, though a small molecule, is restricted partially by BBB. SRS is hypothesized to disrupt BBB locally thereby increasing the lapatinib delivery to brain (75, 76). Hence, resulting in synergistic activity of both agents and resulting in local control improvement as is observed with other TKIs (VEGR TKI for RCC and BRAF/MEK inhibitors for Melanoma) (53, 71, 73, 77–79). Moreover, it can also be observed in the results of Kim et al., demonstrating a significant increase in complete responses with concurrent delivery of lapatinib with SRS (35 vs. 11%, *P* = 0.008) (43). This response rate is comparatively better in comparison to other studies that lacked the use of SRS or any local therapy in addition to lapatinib. Objective response rates between 33.3% to 57% was revealed for lapatinib plus capecitabine management (56, 80, 81). LANDSCAPE phase trial had reported only two cases of complete response [ORR: 24/42 (57%); CR: 2; PR: 22; SD: 15; PD: 3], Shawky, H. and H. Tawfik reported 0 cases [ORR:7/21 (33.3%); CR: 0; PR: 7], and Kaplan et al. also reported 0 cases of complete response [ORR: 14/38 (36.8%), CR: 0; PR: 14; SD:12; PD: 12] (56, 80, 81). Moreover, SRS use in the study by Kaplan et al. was significant for overall survival advantage (MST: 20.3 vs. 11 months, p = 0.007) (56). These outcomes emphasize the use of lapatinib in combination with SRS in order to allow more time and dose for lapatinib activity in the brain, and obtain synergistic activity.

Kim et al. revealed no difference in distant control for SRS plus lapatinib compared to SRS alone (43). This result in line with the data reported in several retrospective studies that involved other TKIs use for RCC (VEGFR TKIs/mTORi) and melanoma BMs (BRAFi/MEKi) (71–74). In contrast, patients with breast cancer (HER2+/HER-) had obtained better distant control with the use of targeted agents with SRS in the study by Johnson et al. (median 10 vs. 5 months, *p* = 0.002) (53). Kotecha et al. had also shown improvement in distant control with the use of BRAFi plus SRS (12-m CI of DF: 68 vs. 95%, p = 0.03) (82). Improved distant control with use of TKIs was revealed by Miller et al. (12-month cumulative incidence of distant failure: 9.2 vs. 18.3%, p = 0.08) for patients with upfront local therapy with/without WBRT (42). The 12-months cumulative incidences were 24.8% for luminal B (95% CI: 17.0–32.5%), and 17.3% for HER2 + (95% CI: 9.5–25.1%). For patients receiving SRS as upfront therapy without WBRT, the 12-month cumulative incidence of distant failure increased to 38% for luminal B (95% CI: 13–62%), and 53% for HER21 (95% CI: 26–81%). Miller et al. also identified that the use of WBRT significantly reduced the 12-month cumulative incidences of distant failure (12m-CI DF: 17.4 vs. 28.4%, p < 0.01) for all patients that also included triple negative and luminal A in addition to luminal B and HER-2 positive BC patients (42). Similarly, significant distant control was reported for VEGFR TKIs and mTOR inhibitors in RCC BM patients (12-mCI: 16.9 vs. 10.5%, p = 0.003) but significance was lost when upfront WBRT was excluded from analysis (26.8 vs. 24.4%, p = 0.150) (83). These outcomes highlight the role of WBRT in enhancing the response rate of lapatinib and decreasing the distant failure rates. Moreover, WBRT plus lapatinib was associated with increased intracranial response rates in a phase I study (79%), indicating better local control as well (38). Therefore, despite the increasing trend of SRS as the sole treatment of BM for this group of patients, the role of WBRT still can’t be ruled out and would need further evaluation given the safety concerns with its use.

Lapatinib is associated with a number of systemic and neurologic side effects such as diarrhea, fatigue, nausea, vomiting, and rash etcetera (27, 37). However, none systemic side effects were reported in all the studies (39–44). Three studies reported radiation necrosis rated for treatment difference (42–44). Miller et al. reported a decrease in RN rates with lapatinib plus SRS use while Kim et al. revealed a lower rate of RN in concurrent lapatinib group as compared to SRS alone (42, 43). Radiation necrosis is a dose-limiting toxicity of SRS occurring in 5 to 10% of patients (54). In a study of nearly 2000 patients undergoing SRS, 15% had experienced RN, and HER-2 amplification was identified as an associated factor (HR 2.05, p = 0.02) along with other histology such as renal, lung adenocarcinoma, ALK/BRAF mutational status (84). In their study, the use of HER2 antibodies (5.9 vs. 7.9%, p = 0.50) or lapatinib (0 vs. 9%, p < 0.01) within 30 days of SRS were not associated with increased 12-month cumulative incidence of RN in the HER2 amplified population. It maybe hypothesized that lapatinib may lower the number of HER-2 oncoproteins thereby reducing the radiosensitivity to SRS that is observed with mutated oncoproteins (ALK/EGFR/BRAF). In line with these data, concurrent lapatinib was not associated with any increased 12-month cumulative incidences of RN in study of Kim et al. (12.5 vs. 7.7%, p = 0.24) (54).

Our study is limited by retrospective nature of the included studies (39–44). Retrospective studies are subject to confounding and tend to have selection bias, recall bias, and misclassification bias (85). These outcomes were also reflected in the results of quality assessment. Only 6 studies were available for inclusion (39–44). Some of the results in our study were also based on outcomes from one or two studies. Moreover, some studies had very limited number of participants (39, 41). Yomo et al. study is limited due to its low number of patients and high cross over (41). Of the total 40 patients, 12 patients from lapatinib based therapy had switched from trastuzumab and later 6 patients had cross over to trastuzumab after using lapatinib. This cross over could have confounded the survival outcome as no survival difference was achieved despite significant brain local control with lapatinib-based therapy. Bartsch et al. study had very few patients receiving lapatinib (39). Its comparison to patients with radiotherapy alone yielded a very low hazard ratio and high standard error due the huge difference between the survival rate. This result has caused the heterogeneity among some results.

### Future Perspective

Lapatinib is the first proven HER-2 targeting tyrosine kinase inhibitor (10). As monotherapy, it has only shown a mild 2.6% to 6% intracranial response (27, 37). In combination with capecitabine response rate was increased between 20% to 57% (37, 80). Response rate was further enhanced in combination with WBRT (79%), and SRS (75%) (38, 43). Lower response rate as monotherapy suggests low bioavailability of the drug in the target tissue. Preclinical and clinical evidence have suggested BBB restriction of lapatinib (69, 70, 86). Two members of ATP-binding cassette (ABC) family of transporters namely P-glycoprotein (P-gp; ABCB1) and breast cancer resistance protein (BCRP; ABCG2) have been implicated in the restriction of several drugs including lapatinib (87–91). Elacridar, an ABCB1 and ABCG2 blocker, was shown to enhance the penetration of lapatinib into the CSF and brain tissue (92). It’s an area that could further enhance the effectivity of lapatinib in the brain. Our results also suggest that combination of lapatinib and trastuzumab enhances the outcome compared to each treatment alone that has also been demonstrated in the case of HER-2 + MBC patients (60–66). In the phase I trial, impressive 79% response was achieved through concurrent use of both agents with WBRT (38). Hence, a combo might further enhance the survival outcome for BCBM patients given the safety concerns are alleviated. On the other hand, SRS has not been tested yet for feasibility with lapatinib and trastuzumab combination. Results of a clinical trial are awaiting in this regard which has combined WBRT or SRS with or without lapatinib in BCBM patients (NCT01622868). EMILIA trial has shown superior efficacy for T-DMI compared to lapatinib plus capecitabine in MBC patients (93). However, recent data suggests the combination of T-DMI and SRS was associated with an increased brain toxicity limiting its use for BCBM patients (94, 95). Therefore, Lapatinib in combination with SRS/WBRT with caution should be evaluated for these patients for further improvement in the survival and quality of life. Nonetheless, overall research regarding the treatment of BCBM patients have not been rigorous. Fares et al. identified several factors impeding the BCBM research such as low number of trials, low accrual numbers, and lack of diversity (96). In addition, these hurdles would also need to be overcome through proper selection of treatment regimens to be investigated. Systematic reviews and meta-analysis such as this could facilitate such selection and assist in optimizing the BCBM research.

## Conclusions

Improvement in survival is observed for HER-2 positive BC patients with BMs being treated with lapatinib-based management. Local brain control was observed with the combination of SRS and lapatinib. Concurrent lapatinib may have better effect as increased intracranial responses were also witnessed. WBRT given in combination with SRS was also shown to have an impact on distant brain control, suggesting a role for WBRT in this group of patients. Lapatinib with SRS was revealed to have lower risk for radiation necrosis in comparison to SRS alone.

## Data Availability Statement

The original contributions presented in the study are included in the article/supplementary material. Further inquiries can be directed to the corresponding author.

## Author Contributions

All authors have contributed equally. All authors contributed to the article and approved the submitted version.

## Funding

This work was supported by the Natural Science Foundation of Shenzhen (no. JCYJ20170307095828424), Shenzhen Health and Family Planning System Research Project (no. SZBC2017024), and the technical research and cultivation project for the youth of Shenzhen People’s Hospital (no. SYKYPY2019029).

## Conflict of Interest

The authors declare that the research was conducted in the absence of any commercial or financial relationships that could be construed as a potential conflict of interest.
